# Hyperlipidemia-Associated Renal Damage Decreases Klotho Expression in Kidneys from ApoE Knockout Mice

**DOI:** 10.1371/journal.pone.0083713

**Published:** 2013-12-30

**Authors:** Cristina Sastre, Alfonso Rubio-Navarro, Irene Buendía, Carmen Gómez-Guerrero, Julia Blanco, Sebastian Mas, Jesús Egido, Luis Miguel Blanco-Colio, Alberto Ortiz, Juan Antonio Moreno

**Affiliations:** 1 Vascular and Renal Research Lab, IIS-Fundación Jiménez Díaz, Autónoma University, Madrid, Spain; 2 Hospital Clinico San Carlos, Madrid, Spain; 3 Spanish Biomedical Research Centre in Diabetes and Associated Metabolic Disorders (CIBERDEM), Spain; Center for Molecular Biotechnology, Italy

## Abstract

**Background:**

Klotho is a renal protein with anti-aging properties that is downregulated in conditions related to kidney injury. Hyperlipidemia accelerates the progression of renal damage, but the mechanisms of the deleterious effects of hyperlipidemia remain unclear.

**Methods:**

We evaluated whether hyperlipidemia modulates Klotho expression in kidneys from C57BL/6 and hyperlipidemic apolipoprotein E knockout (ApoE KO) mice fed with a normal chow diet (ND) or a Western-type high cholesterol-fat diet (HC) for 5 to 10 weeks, respectively.

**Results:**

In ApoE KO mice, the HC diet increased serum and renal cholesterol levels, kidney injury severity, kidney macrophage infiltration and inflammatory chemokine expression. A significant reduction in Klotho mRNA and protein expression was observed in kidneys from hypercholesteromic ApoE KO mice fed a HC diet as compared with controls, both at 5 and 10 weeks. In order to study the mechanism involved in Klotho down-regulation, murine tubular epithelial cells were treated with ox-LDL. Oxidized-LDL were effectively uptaken by tubular cells and decreased both Klotho mRNA and protein expression in a time- and dose-dependent manner in these cells. Finally, NF-κB and ERK inhibitors prevented ox-LDL-induced Klotho downregulation.

**Conclusion:**

Our results suggest that hyperlipidemia-associated kidney injury decreases renal expression of Klotho. Therefore, Klotho could be a key element explaining the relationship between hyperlipidemia and aging with renal disease.

## Introduction

Chronic kidney disease (CKD) has features consistent with accelerated aging, such as augmented atherosclerosis [1]. Abnormalities in lipid and lipoprotein metabolism frequently accompany renal disease and are thought to be involved in the pathogenesis of renal injury [2], [3]. Hyperlipidemia is associated with formation of oxidized low-density lipoprotein (ox-LDL). Accumulation of ox-LDL has been reported in the circulation and renal interstitium of patients with CKD and end stage-renal disease [4]. In the kidney, these atherogenic lipoproteins are cytotoxic, promote macrophage recruitment, enhance oxidative stress, and promote pro-inflammatory cytokines expression [5], [6], changes that may compromise renal function [7]–[9].

Klotho is a kidney protein that plays a pivotal role in regulating aging and the development of age-related diseases [10], [11]. Klotho knockout mice exhibit multiple aging-like phenotypes, including atherosclerosis, osteoporosis, emphysema and infertility [10]. By contrast, *in vivo* Klotho gene delivery can ameliorate vascular endothelial dysfunction, increase nitric oxide production, reduce elevated blood pressure, and prevent fibrosis [12]. Klotho antiaging properties include modulation of Ca2+/Pi homeostasis [13], [14], antioxidant effects [15], suppression of insulin-like growth factor I signaling [11], protection of endothelium through induction of endothelial nitric oxide synthesis, inhibition of tumor necrosis factor (TNF)-α-induced nuclear factor (NF)-κB activity and expression of endothelial adhesion molecules [16], [17]. Kidney Klotho is downregulated in sustained hypertension, diabetes mellitus, and CKD models [18] as well as in experimental ischemia-reperfusion-induced or nephrotoxic acute kidney injury (AKI) [19], [20]. Furthermore, decreased expression of Klotho mRNA and protein was confirmed in human CKD [21].

To date, the regulatory mechanisms of Klotho gene expression remains poorly understood. Klotho expression is reduced by acute inflammatory stress *in vivo* [22], and also by angiotensin II and oxidative stress in tubular cells both in cell culture and *in vivo* [23]–[26]. Statins and Rho kinase inhibitors prevented angiotensin-induced downregulation of Klotho in cultured cells [24]. In addition, pro-inflammatory cytokines TWEAK (Tumor necrosis factor like weak inducer of apoptosis) and TNF-α may also reduce renal Klotho expression throughout NF-κB activation [19].

Because renal failure and hyperlipidemia are both associated with accelerated aging features, such as atherosclerosis, oxidative stress and endothelial dysfunction, characteristics also observed in Klotho knockout mice [10]–[12], [25], in this study we investigated the impact of hyperlipidemia-associated renal damage on Klotho expression in kidneys from control and hyperlipidemic Apolipoprotein E (ApoE)-knockout (ApoE KO) mice, a widely used model to study the effect of early onset hyperlipidemia on renal injury [27], [28]. Furthermore, we analyzed the effect of ox-LDL on Klotho expression in renal tubular epithelial cells as well as the signaling pathways involved in the modulation of klotho expression by these modified lipoproteins.

## Materials and Methods

### Animal Model

One experimental design studied early renal lesions. Sixteen male C57BL/6 mice (Harlan laboratories) and sixteen male ApoE knockout mice (12 weeks of age; Jackson Laboratories) were randomly divided in two groups fed either a normal (ND, 5.0% fat, 0.05% cholesterol) or a high fat hyperlipidemic diet (HC, 21.2% fat, 0.15% cholesterol) +16.7% proteins) for 5 weeks. A second experimental design studied advanced renal lesions. Sixteen male C57BL/6 mice and sixteen male ApoE knockout mice (16 weeks of age) were fed a ND or HC for 10 weeks. Blood was collected in serum tubes and stored at −80°C until used. Cholesterol was tested in serum samples (Thermo Trance). Anesthetized mice were saline-perfused. One kidney was snap-frozen in liquid nitrogen for RNA and protein studies, and the other kidney was fixed in 4% paraformaldehyde, embedded in paraffin and used for immunohistochemistry.

### Renal Histophatologic Studies and Immunochemistry

To assess histological changes, paraffin-embedded kidneys were cross-sectioned into 5 µm thick pieces and Masson trichrome stained. Samples were examined by an outside pathologist (JB) blinded to the nature of the samples. Tissue sections were scored according to the extent of glomerular changes (hypertrophy, hypercellularity, mesangial proliferation, ischemia, sclerosis, foam cells and fibrosis), tubular (loss of brush border, vacuolization, desquamation, tubular dilation) and interstitial damage (interstitial fibrosis and interstitial infiltrate) in a semiquantitative scale from 0 to 3, and results from each item were added to yield the tubular, glomerular or interstitial injury score.

Immunohistochemistry was carried out as described previously in paraffin embedded tissue sections 5 µm thick [19]. Primary antibodies were rat polyclonal anti-F4/80 antigen (1∶50, Serotec), goat polyclonal anti-monocyte chemotactic protein-1 (MCP-1) (1/100, Santa Cruz Biotechnology, Santa Cruz, California) and rabbit polyclonal anti-Regulated on Activation, Normal T Cell Expressed and Secreted (RANTES) (1/200, Antibodies-online, Aachen, Germany). Sections were counterstained with Carazzi‘s hematoxylin. Negative controls included incubation with a nonspecific Ig of the same isotype as the primary antibody. Results were expressed as percentage positive area versus total area of RANTES and MCP-1 staining in 20 randomly selected fields (x100) per sample. Quantification of F4/80 positive-stained cells was made by determining the total number of positive cells/total number of cells in 20 randomly chosen fields (x100) using Image-Pro Plus software (Media Cybernetics, Silver Spring, MD). Samples from each animal were examined in a blinded manner. Lipids were detected by Oil red O staining as previously reported [6].

For immunofluorescence studies, murine proximal tubular epithelial cells (MCT) and murine distal tubular epithelial cells (NP-1) were plated onto Labtek slides, fixed in 4% paraformaldehyde and permeabilized in 0.2% Triton X-100/PBS, washed in PBS, and incubated with rabbit polyclonal anti-Klotho (1∶100. Calbiochem, La Jolla, California), followed by Alexa 488 secondary antibody (1∶200, Invitrogen, Eugene, Oregon, USA) or Alexa 633 secondary antibody (1∶200, Invitrogen, Eugene, Oregon, USA). Nuclei were counterstained with propidium iodide or 4′-6-diamidino-2-phenylindole (DAPI). Klotho-expressing tubular cells in kidney from normal mice were detected using the anti-Klotho antibody with secondary Alexa Fluor 633 and then stained with the proximal tubule marker, fluorescein-conjugated *Tetranogolobus lotus* (1∶33, Sigma).

### Time of Flight Secondary Ion Mass Spectrometry (TOF-SIMS)

Samples were kept at -80°C until 10-µm slices were cut using a cryostat (CM1900; Leica) at a constant temperature of −25°C. Tissue sections were deposited onto a stainless steel plate (15-7PH; Goodfellow) and stored again at −80°C. After drying under a pressure of a few hPa for 15 min, they were directly analyzed in a TOF-SIMS V mass spectrometer (IonTof, Germany) fitted with a bismuth cluster ion source located at the Parque Cientifico de Barcelona, Spain. The primary ions impinge the surface of the tissue section with a kinetic energy of 25 keV. The primary ion dose was between 4.7×10^11^ and 10^12^ ions/cm^2^. The secondary ions were extracted with 2 keV energy and postaccelerated to 10 keV just before hitting the detector surface (single-channel plate followed by a scintillator and a photomultiplier). A low-energy electron flood gun was activated to neutralize the surface during the analysis. The effective ion flight path is ∼2 m using a reflectron, and the mass resolution is ∼6,300 (full width at half-maximum) at a mass-to-charge ratio (m/z) of 35 and 10,000 at 795.7 m/z. Mass calibration and ion peak identifications were performed as previously described [29]. Color scales correspond to the interval (0, maximal number of counts in a pixel).

### Isolation and Oxidation of LDL

Human LDL were isolated from a fresh plasma pool by sequential ultracentrifugation (2 hour at 405,000 *g* and 4°C) immediately after separation of plasma at density between 1.019 and 1.063 and after very low density lipoprotein (VLDL) isolation at d<1.019 using a Beckman model LE-70 ultracentrifuge with a Type NVT65 rotor (Beckman). The LDL fraction was dialyzed for 24 hours at 4°C with 3 exchanges of PBS, containing 0.01% EDTA for the first and only PBS for the 2 other exchanges. LDL protein content was determined by using the method of Bradford. LDL oxidation was carried out by incubating 200 µg/mL LDL protein with 5 µmol/L CuSO_4_ in 1.0 mL PBS medium at 37°C for 120 minutes. The reaction was stopped by adding 100 mmol/L EDTA at 4°C and immediately dialyzed to eliminate EDTA. Oxidized-LDL were filtered and stored at 4°C in nitrogen atmosphere.

### Cell Culture

MCT and NP-1 cells were cultured in RPMI 1640 or DMEM, respectively, supplemented with decomplemented fetal bovine serum (10%), glutamine (2 mmol/l), and penicillin/streptomycin (100 U/ml; LONZA, Verviers, Belgium) in 5% CO_2_ at 37 °C. Both cell lines were originated from the kidneys of SJL mice in the University of Pennsylvania and obtained from Eric G Neilson and Frank Strutz, respectively [30]. Cell viability experiments were performed by propidium iodide flow cytometry as previously reported [31]. For inhibition experiments, cells were pre-treated for 1 hour with inhibitors of Phosphoinositide 3-Kinase (PI3K) (LY294002, 20 µM, Sigma), NF-kB (Parthenolide, 1 µM, Sigma), Jun N-terminal kinase (JNK) (SP600125, 10 µM, Stressgen Bioreagents), p38 (SB203580, 10 µM, Stressgen Bioreagents), extracellular signal-regulated kinase (ERK) (PD98059, 50 µM, Stressgen Bioreagents), and then stimulated with ox-LDL (25 µg/mL).

### ELISA

Concentrations of murine MCP-1 (BD Pharmingen), interleukine 6 (IL-6) (BD Pharmingen) and RANTES (Bender MedSystems) in the supernatants of the cell cultures were determined by ELISA, following the manufacturer’s instructions.

### RNA Extraction and Real-time PCR

Total RNA from kidneys or cultured cells was obtained by Trizol method (Invitrogen, Carlsbad, CA, USA) and reverse-transcribed with High Capacity cDNA Archive Kit and real-time PCR was performed on a ABI Prism 7500 PCR system (Applied Biosystems, Foster City, California) using the DeltaDelta Ct method. Expression levels are given as ratios to Glyceraldehyde 3-phosphate dehydrogenase (GADPH). Predeveloped primer and probe assays were obtained for murine GADPH, RANTES, TNF-alpha, MCP-1, IL-6 and Klotho (Applied Biosystems).

### Western Blot

Cell samples or tissues were homogenized in lysis buffer (50 mM TrisHCl, 150 mM NaCl, 2 mM EDTA, 2 mM EGTA, 0.2% Triton X-100, 0.3% NP-40, 0.1 mM PMSF, and 1 µg/ml pepstatin A) and then separated by 10% SDS-PAGE under reducing conditions. After electrophoresis, samples were transferred to PVDF membranes (Millipore, Bedford, Massachusetts), blocked with 5% skimmed milk in PBS/0.5% vol/vol Tween 20 for 1 h, washed with PBS/Tween, and incubated with rabbit polyclonal anti-Klotho (1∶500, Calbiochem, La Jolla, California), IκB-alpha (sc-371, Santa Cruz), β-actin (sc-47778, Santa Cruz), p-ERK (sc-7383, Santa Cruz), and ERK (sc-154, Santa Cruz). Antibodies were diluted in 5% milk PBS/Tween. Blots were washed with PBS/Tween and incubated with appropriate horseradish peroxidase-conjugated secondary antibody (1∶2000, Amersham, Aylesbury, UK). After washing with PBS/Tween the blots were developed with the chemiluminescence method (ECL, Amersham). Blots were then probed with mouse monoclonal anti-α-tubulin antibody (1∶2000, Sigma), and levels of expression were corrected for minor differences in loading. Quantification was expressed as arbitrary densitometric units (AU).

### Measurement of NADPH-dependent ROS Production and Detection of Superoxide Anion

A lucigenin-enhanced chemiluminescence assay [32] was used to determine the nicotinamide adenine dinucleotide phosphate-oxidase (NADPH)-dependent reactive-oxygen species (ROS) production activity in MCT cells lysates. The reaction mixture comprised 50 mM phosphate buffer containing 1 mM EGTA (pH 7.0), 5 µM lucigenin, and 0.1 mM NADPH. Chemiluminescence was measured for 5 min after the addition of NADPH and recorded in a Sirius luminometer (Berthold Detection System). ROS production was determined from the ratio of the relative light units to the total protein levels and expressed as fold vs. basal. Dihydroethidium (DHE; Molecular Probes, Invitrogen) was used to evaluate *in situ* levels of superoxide as described previously [33].

### Statistical Analysis

Statistical analysis was performed using SPSS 11.0 statistical software. Significance at the p<0.05 level was assessed by Student’s t-test for two groups of data and ANOVA for three or more groups. In vitro experiments were replicated three times for each incubation period. Pearson differences were considered significant at a value of p<0.05.

### Ethics Statement

The housing and care of animals and all the procedures carried out in this study were strictly in accordance with the Directive 2010/63/EU of the European Parliament and were approved by the Institutional Animal Care and Use Committees of IIS-Fundacion Jimenez Diaz.

## Results

### Hyperlipidemia Increases Kidney Lipid Deposits and Aggravates Renal Damage

**Figure 1:**
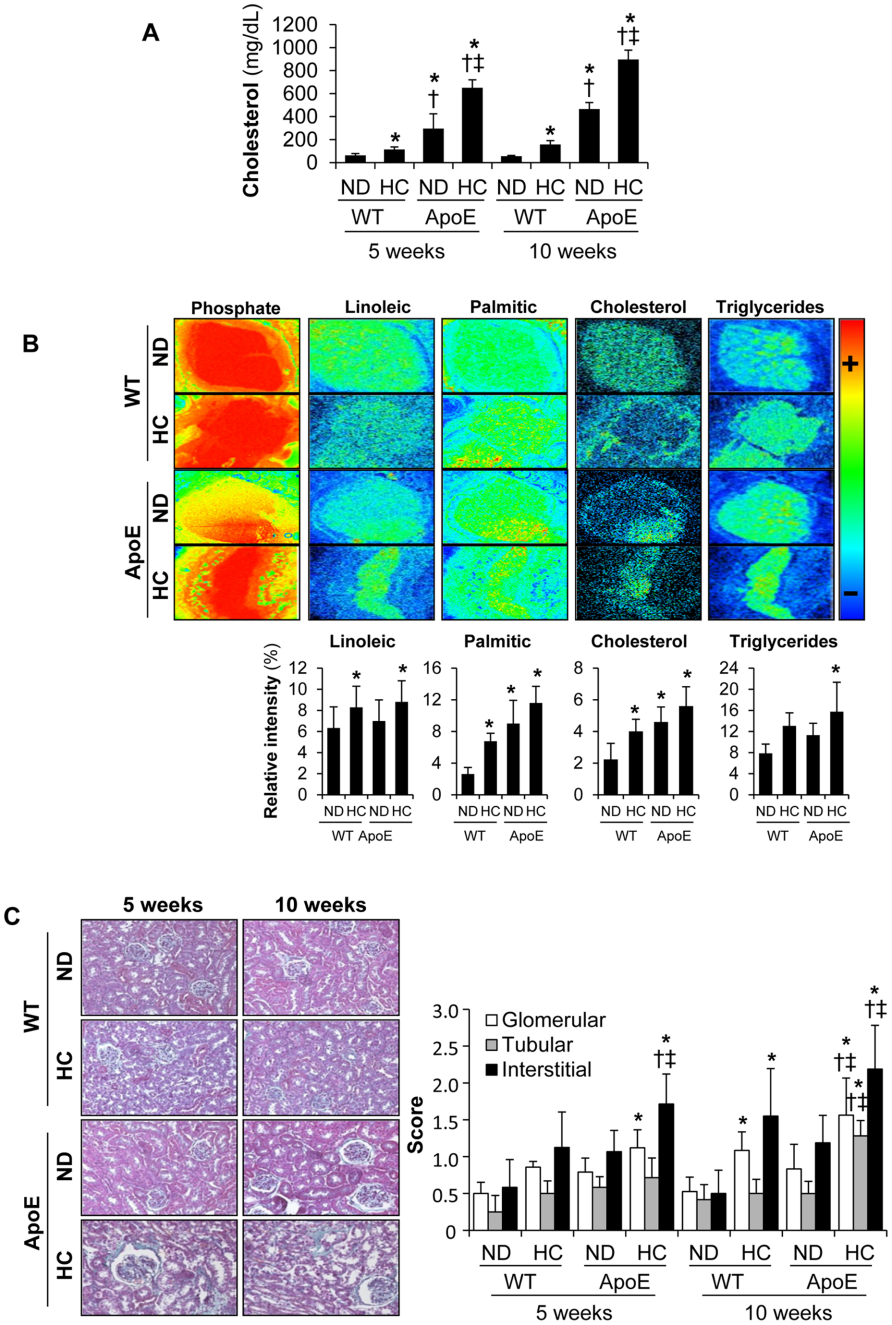
Murine model of renal damage induced by hyperlipidemia. (**A**) Serum cholesterol levels from C57BL/6 (WT) and ApoE knockout (ApoE) mice fed a normal chow diet (ND) or a hyperlipidemic diet (HC) for 5 or 10 weeks. (**B**) Semiquantitative assessment and representative secondary ion images under the irradiation of bismuth cluster showing lipid content in kidneys from mice at 10 weeks of dietary intervention. In order to normalize tissue size variation, metabolite measurements were expressed as % relative intensity = ratio between the metabolite area and total tissue area (phosphate area) (**C**) Representative Masson trichrome staining in mouse kidneys and semiquantitative assessment of lesions in glomerular, tubular and interstitial compartments. Original magnification x 100. *p<0.05 vs WT with ND in each time-period, † p<0.05 vs WT with HC in each time-period, ‡ p<0.05 vs ApoE knockout mice with ND in each time-period. Values shown are mean±SD, n = 8 per group.

As expected, serum cholesterol levels were much higher in ApoE KO mice than in C57BL/6 wild type mice (WT) (**Figure 1A**). HC diet induced an increase in total cholesterol in both WT and ApoE KO mice, but hyperlipidemia was more pronounced in ApoE KO mice. In order to determine whether hyperlipidemic mice show enhanced renal lipid accumulation, we performed metabolomic TOF-SIMS imaging analysis [29]. Renal samples subjected to TOF-SIMS analysis had a strong bias toward hydrophobic molecules, displaying high-resolution images of the most abundant lipids and lipid derivatives present on the sample surface. Kidneys from hyperlipidemic ApoE KO mice fed a HC diet showed an increased accumulation of cholesterol and triglycerides as compared with WT mice fed a ND (**Figure 1B**). Furthermore, HC increased kidney palmitic acid (C16∶0) and linoleic acid (C18∶2) in both WT and ApoE KO mice. Finally, we also observed increased neutral lipid accumulation, as determined by oil red O staining, mainly in glomeruli from ApoE KO mice fed a HC for 10 weeks; whereas a mild staining or no glomerular staining was observed in ApoE KO mice on ND or WT mice, respectively (**Fig. S1**). All together, these data strongly indicated that there were excessive amounts of lipid deposits in the kidneys of ApoE KO mice fed a HC.

Standard histological analysis showed increased glomerular, tubular and interstitial renal damage in hyperlipidemic ApoE KO mice after HC as compared to WT fed either ND or HC diet (**Figure 1C**). The main renal findings in ApoE KO mice fed HC included accumulation of foam cells in glomeruli, increased mesangial proliferation and interstitial inflammatory infiltrates. We also observed tubular dilation, vacuolization and loss of brush border in tubular epithelium of these hyperlipidemic mice. Furthermore, ApoE KO mice fed HC presented significantly more severe glomerular, tubular and interstitial renal damage, as compared with ApoE KO mice fed a ND diet. All these differences increased as duration of HC augmented.

### Increased Renal Inflammation and Oxidative Stress in Hyperlipidemic Mice

**Figure 2:**
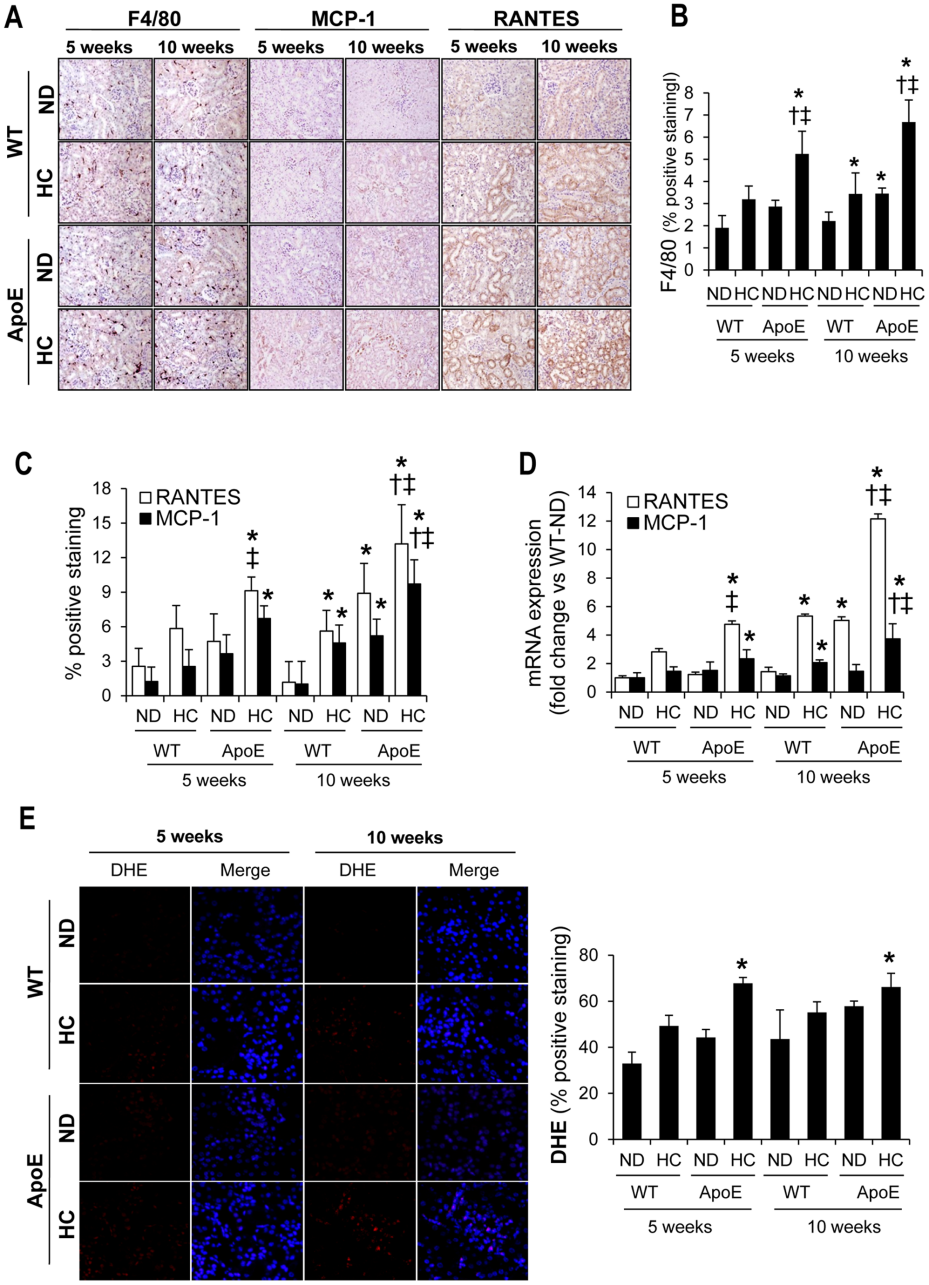
Inflammatory response and oxidative stress in murine kidneys. Representative inmunohistochemistry (**A**) and semiquantitative assessment (**B–C**) of renal macrophage infiltration (F4/80) and inflammatory cytokines (RANTES and MCP-1) in kidneys from C57BL/6 (WT) and ApoE knockout (ApoE) mice fed normal chow-(ND) or hyperlipidemic-diet (HC) for 5 or 10 weeks. Original magnification x 100. (**D**) Expression of MCP-1 and RANTES, as determined by real time RT-PCR, in kidneys from all the studied groups. (**E**) Representative DHE staining and semiquantitative DHE assessment in kidneys from all mice groups (magnification x 200). *p<0.05 vs WT with ND in each time-period, † p<0.05 vs WT with HC in each time-period, ‡ p<0.05 vs ApoE knockout mice with ND in each time-period. Values shown are mean±SD, n = 8 per group.

An increased tubulointerstitial inflammation, characterized by increased number of interstitial F4/80-positive macrophages, was observed in ApoE KO mice after HC consumption as compared with WT fed on either ND or HC or ApoE KO mice fed ND, independently of the time-period. In WT mice, HC consumption for 10 weeks also augmented macrophage infiltration; however no significant differences were observed after 5 weeks of HC intake (**Figure 2A–B**). Hyperlipidemic ApoE KO mice fed a HC diet for 5 or 10 weeks presented an increased presence of inflammatory chemokines RANTES and MCP-1, as compared with WT fed either ND or HC (**Figure 2A and 2C**). HC also increased the expression of both chemokines in WT mice, but only after 10 weeks of intervention. At this time, ApoE KO mice on ND presented higher RANTES and MCP-1 expression than WT mice on ND. Determination of RANTES and MCP-1 mRNA expression by quantitative RT-PCR analysis confirmed these results (**Figure 2D**). Furthermore, ApoE KO mice fed a HC diet for 5 or 10 weeks presented higher superoxide anion levels, as determined by DHE staining, indicating the presence of an increased oxidative stress in these animals (**Figure 2E**).

### Klotho mRNA and Protein are Decreased in Hyperlipidemic Renal Injury

**Figure 3:**
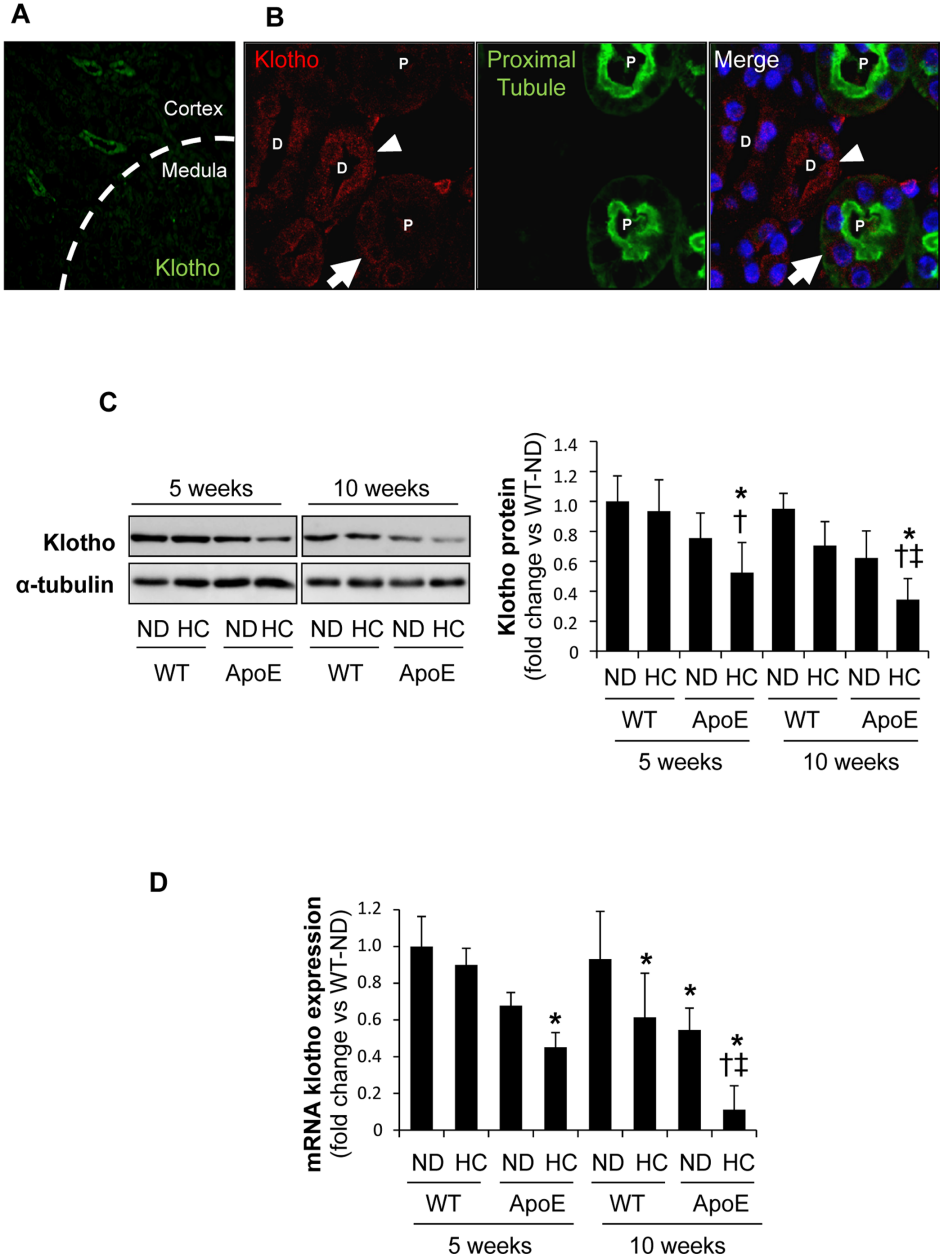
Decreased Klotho expression in kidneys from hyperlipidemic mice. (A) Localization of renal Klotho using anti-Klotho with secondary Alexa Fluor 488–conjugated antibody (green) in a normolipidemic WT mice. (**B**) Klotho-expressing tubular cells in kidney from WT mice were detected using anti-Klotho with secondary Alexa Fluor 633–conjugated antibody (red) and then stained with proximal tubule marker FITC-Tetranogolobus lotus (green). Nuclei were stained with DAPI (blue). Arrows and arrowheads identify Klotho expression in proximal and distal nephron tubules, respectively. (**C**) Total Klotho kidney protein expression, as determined by Western-blot in kidneys from C57BL/6 (WT) and ApoE knockout (ApoE) mice fed normal chow-(ND) or hyperlipidemic-diet (HC) for 5 or 10 weeks. (**D**) Klotho mRNA expression in kidneys as determined by real time RT-PCR. *p<0.05 vs WT with ND in each time-period, † p<0.05 vs WT with HC in each time-period, ‡ p<0.05 vs ApoE knockout mice with ND in each time-period. Values shown are mean±SD, n = 8 per group.

In normolipemic WT mice, Klotho expression was mainly detected in both proximal and distal tubules from the renal cortex, whereas no positive staining was observed in the renal medulla (**Figure 3A–B**). A non-significant trend toward decrease in renal Klotho content was observed in WT mice fed HC diet, as determined by Western blot (**Figure 3C**). Interestingly, HC caused a significant reduction in the renal expression of Klotho in hyperlipidemic ApoE KO, and this effect was markedly observed after 10 weeks of intervention. The same results were obtained when we determined Klotho mRNA expression by quantitative RT-PCR, suggesting that hyperlipidemia could downregulate renal Klotho expression (**Figure 3D**).

### Oxidized-LDL Decrease Klotho Expression and Increase Proinflammatory Cytokines and Oxidative Stress in Cultured Renal Tubular Cells

**Figure 4:**
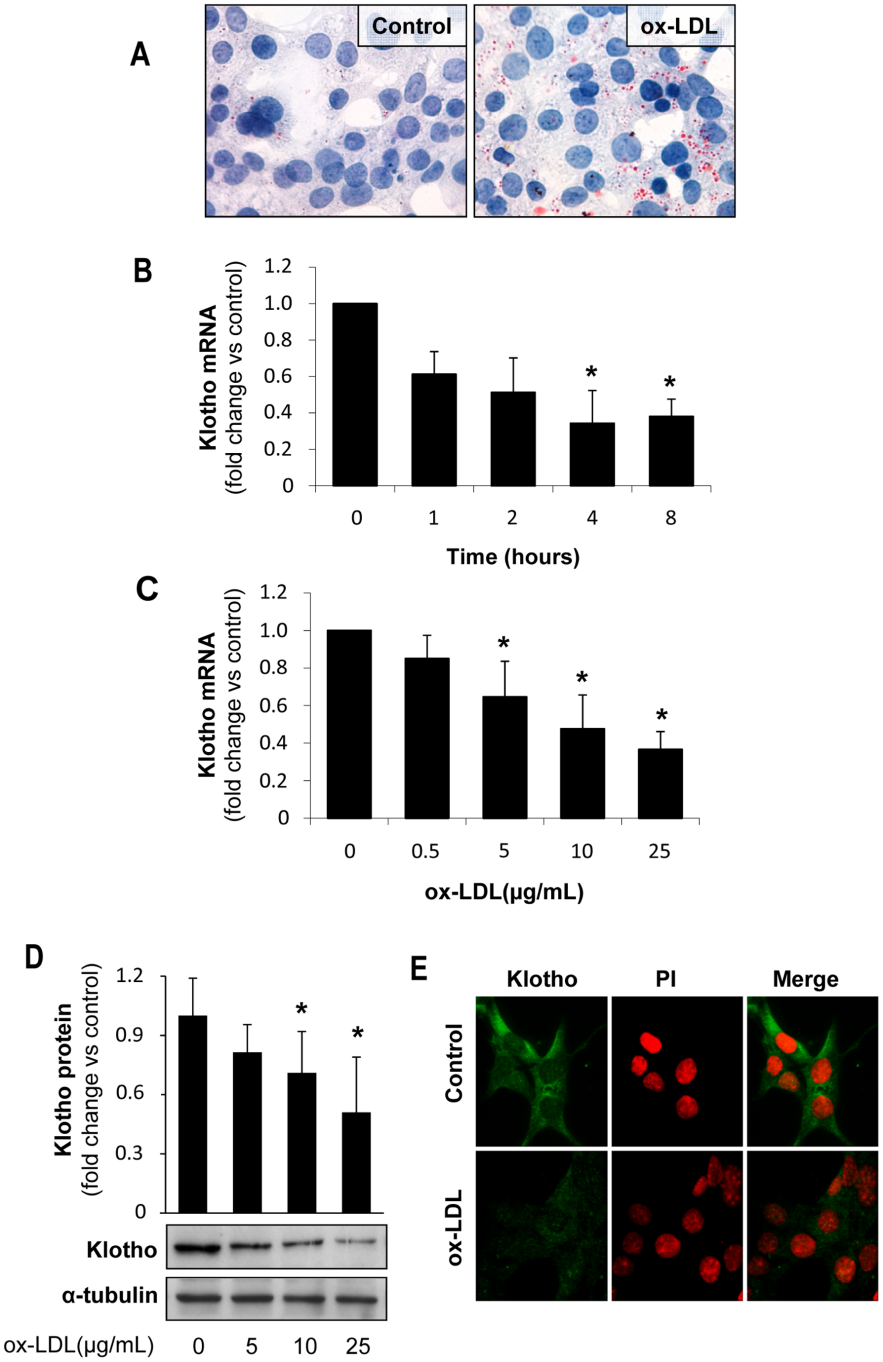
Oxidized LDL decrease Klotho expression in cultured tubular cells. (**A**) Oil-Red-O staining in murine proximal tubular cells (MCT) showing increased lipid accumulation after 24 h incubation with ox-LDL (25 µg/mL). Ox-LDL decreases Klotho mRNA expression, as determined by quantitative RT-PCR, in a time (**B**) and dose-dependent manner (**C**) in proximal tubular cells (MCT). Mean±SD of three independent experiments. *p<0.05 vs control. Klotho protein expression, as determined by Western blot (**D**) and confocal microscopy (**E**), in MCT treated with ox-LDL (25 µg/mL) for 24 h. Indirect immunofluorescence using anti-Klotho with secondary Alexa Fluor 488–conjugated antibody (green). Nuclei were stained with propidium iodide (PI, red). Images are representative of three independent experiments.

Hyperlipidemia is accompanied by formation of ox-LDL, leading to increased oxidative stress and inflammatory cytokine production, changes that could modulate Klotho expression [22]–[26]. Therefore, to analyze the mechanisms involved in the modulation of Klotho by hyperlipidemia *in vivo*, we incubated cultured renal cells in presence of ox-LDL. Since Klotho is mainly produced by proximal and distal tubules in the mouse kidney, our *in vitro* studies were conducted in both mouse proximal (MCT) and distal (NP-1) tubular epithelial cells. Cells were stimulated with different concentrations of ox-LDL (0–50 µg/mL) based on viability dose–response studies (**Figure S2**). Oil red staining revealed that MCTs were able to uptake ox-LDL, as determined by increased neutral lipid accumulation (**Figure 4A**). Importantly, ox-LDL decreased Klotho mRNA expression in a time- and dose-dependent manner (**Figure 4B–C** **)**. Moreover, ox-LDL also decreased Klotho protein, as reported by Western blot and immunofluorescence in MCT (**Figure 4D–E**). Similar results were observed in NP-1 cell cultures (**Fig. S3**).

**Figure 5:**
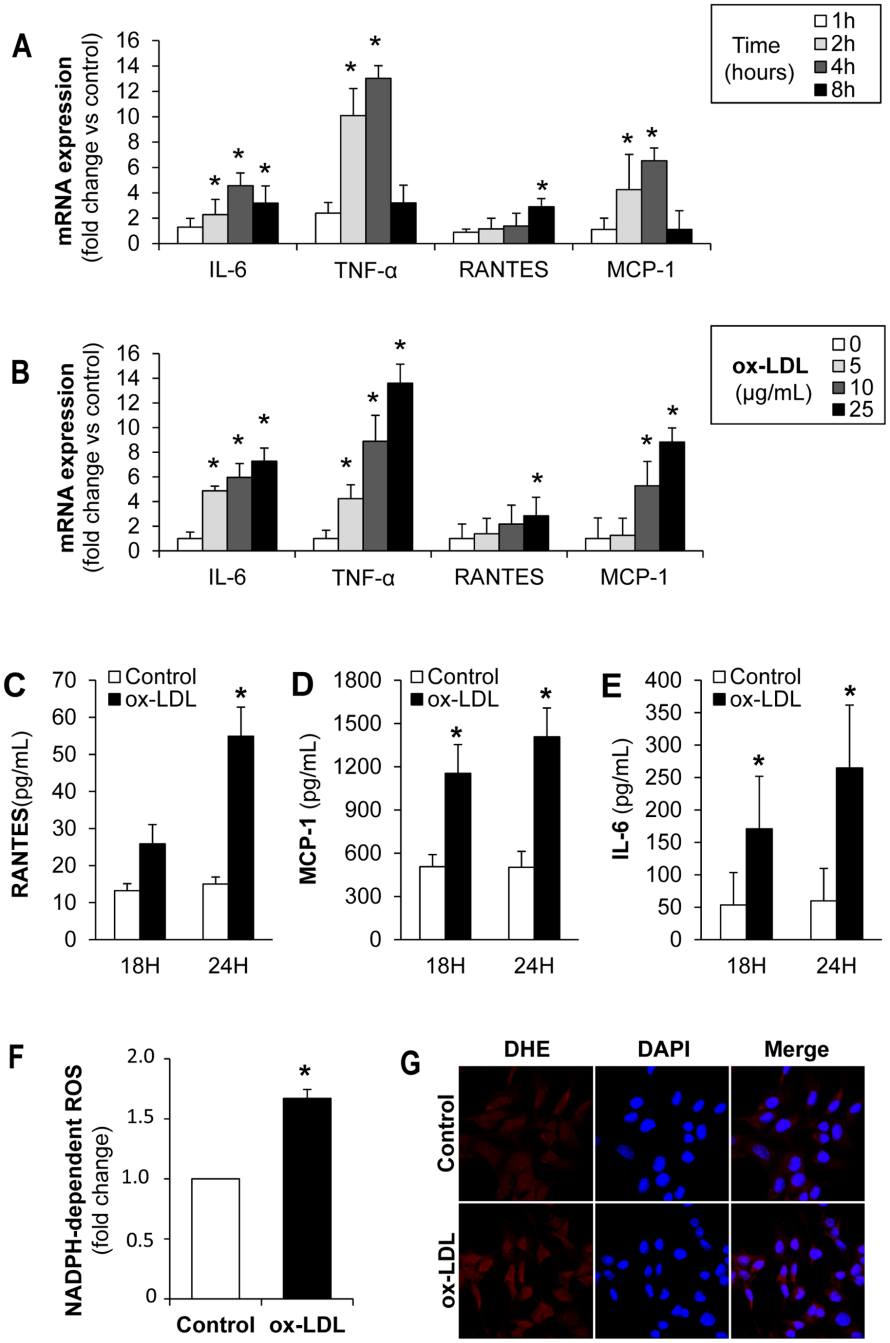
Oxidized-LDL stimulates expression and secretion of pro-inflammatory chemokines and ROS production by proximal tubular MCT cells. (**A**) Quantitative RT-PCR analyses of IL-6, TNF-α, RANTES and MCP-1 in MCT stimulated with ox-LDL (25 µg/mL) at different time points. (**B**) Quantitative RT-PCR analyses of IL-6, TNF-α, RANTES and MCP-1 in MCT stimulated with ox-LDL at different doses (0–25 µg/mL). Values for chemokines were normalized to GAPDH expression and results are expressed as fold-change vs control. (**C–E**) Secretion of RANTES, MCP-1 and IL-6 (at 18 and 24 h) in MCT treated with 25 µg/mL ox-LDL. Supernatants were tested by ELISA. (**F**) NADPH-dependent ROS production in MCT cells treated with ox-LDL (25 µg/ml) for 24 h. (**G**) Representative DHE staining of MCT cells under basal conditions and stimulated with ox-LDL (25 µg/ml) for 24 h. Mean±SD of 3 independent experiments. *p<0.05 vs control.

Because inflammation and oxidative stress have been reported to decrease renal Klotho expression, we analyzed whether ox-LDL promotes the activation of these pathological pathways in tubular epithelial cells. The addition of ox-LDL to MCT cell cultures changed their quiescent epithelial phenotype into a pro-inflammatory one, characterized by increased IL-6, RANTES, MCP-1 and TNF-α gene expression (**Figure 5A–B**). ELISA analysis also showed increased secretion of soluble RANTES, MCP-1 and IL-6 proteins in ox-LDL stimulated cells (**Figure 5C–E**). Nicotinamide adenine dinucleotide phosphate (NADPH) oxidase activation is a major source of reactive oxygen species (ROS) in renal cells [34]. Therefore, we measured ROS generation after ox-LDL treatment in tubular cells. As expected, ox-LDL increased ROS production mediated by NADPH oxidase in MCT (**Figure 5F**). Furthermore, exposure to ox-LDL augmented intracelular superoxide anion levels in MCT, as determined by DHE staining (**Figure 5G**).

### Regulation of Klotho Expression by ox-LDL in Cultured Tubular Cells

**Figure 6:**
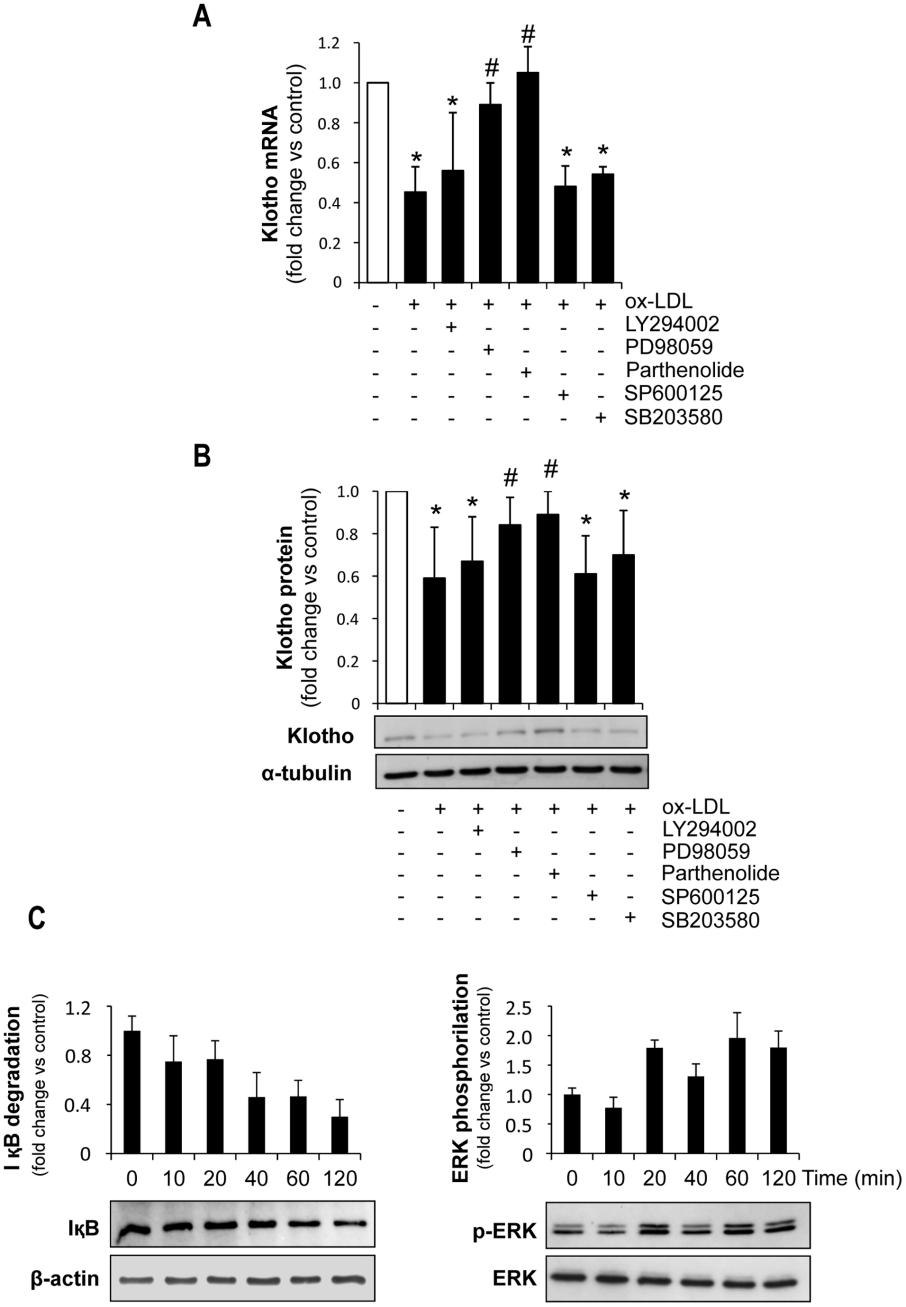
Effect of signaling pathway inhibitors on ox-LDL-induced Klotho downregulation in proximal tubular cells. Pretreatment of MCT with parthenolide (1 µM) or PD098059 (50 µM) for 1 hour attenuates the decrease in Klotho mRNA (**A**) and protein (**B**) induced by ox-LDL (25 µg/mL). (**C**) Treatment with ox-LDL (25 µg/mL) promotes IκB degradation (left panel) and ERK1/2 phosphorylation (right panel) in MCT cells. Cell lysates were analyzed by Western blot for IκB and phospho-ERK 1/2. Each blot was stripped and reprobed with beta-actin and anti-ERK antibody, respectively. Mean±SD of 3 independent experiments. *p<0.05 vs control, # p<0.05 vs ox-LDL.

Oxidized-LDL modulate gene expression by different signaling pathways [35]–[39]. To gain insight into the molecular mechanisms of Klotho downregulation by ox-LDL in tubular cells, the effect of inhibitors of distinct signaling pathways was further analyzed in MCT cultures. As shown in **Figure 6**, inhibitors of PI3K/Akt (LY294002), JNK (SP600125) and p38 (SB203580) did not affect the ability of ox-LDL to decrease Klotho mRNA and protein expression. In contrast, inhibitors of NF-κB (Parthenolide) and ERK (PD98059) restored the mRNA and protein expression levels of Klotho in ox-LDL incubated cells (**Figure 6A–B**). As shown in **Figure 6C**, the NF-κB and ERK pathways were activated by ox-LDL in MCT. Therefore, both NF-κB and ERK signaling pathways are involved in ox-LDL-induced decrease of Klotho expression in tubular cells.

## Discussion

In the present study, we have demonstrated that renal Klotho expression is decreased in hypercholesterolemic ApoE-KO mice fed a high-fat diet. Furthermore, our results suggest that ox-LDL could be related to the reduction of Klotho expression in tubular epithelial cells. Finally, we have identified NF-κB and ERK as the signaling pathways involved in the ox-LDL mediated Klotho decrease.

Klotho is a membrane protein highly expressed in the kidney that binds to FGF23, a hormone that induces phosphate excretion into urine and regulates vitamin D synthesis. Klotho has been also found in a soluble form, in human plasma and cell culture supernatants. The secreted Klotho protein increases endothelium-dependent nitric oxide synthesis and confers resistance to oxidative stress [15]. As a result, lack of Klotho in mice results in accelerated aging and development of age-related systemic disorders, such as atherosclerosis [10], [40]. Atherosclerosis is frequently found in subjects with CKD, increasing the morbidity and mortality of these patients [41], [42]. However, the physiological mechanisms involved in the association of renal failure and atherosclerosis remains to be elucidated. Recently, Klotho has been proposed to play an important role in this association. Thus, a decreased renal Klotho expression was reported in an experimental model of accelerated atherosclerosis in mice with CKD [43]; but the intrinsic factors regulating Klotho in this pathological condition are unknown. In this article we provide evidence for a role of hyperlipidemia in attenuation of Klotho expression.

Hyperlipidemia, specifically hypercholesterolemia, plays an important role in the induction of both vascular and renal injury [2], [3] and is an independent risk factor for CKD progression [44]. In this context, ApoE knockout mouse fed a high-fat diet, which displays massive cholesterol accumulation, is a well-accepted model for studying the effects of early onset hyperlipidemia on renal damage [27], [28]. Renal pathophysiological alterations in this model included lipid accumulation, macrophage infiltration, glomerular infiltration with foam cells and lipid deposits, and expanded mesangium, consistent with previous reports [6], [27]. Previous studies in hyperlipidemic rats with non-insulin-dependent diabetes [45] or hypercholesterolemic uremic atherosclerotic ApoE KO mice reported a decreased renal *klotho* gene expression [43]. However, these studies did not specifically address whether hyperlipidemia-associated renal damage modifies Klotho expression. In our study, we observed that renal Klotho mRNA and protein expression decreased as hyperlipidemia increased. Thus, hyperlipidemic ApoE KO mice fed a HC diet showed the lowest Klotho mRNA and protein values whereas normolipidemic WT on ND had the highest ones.

In our study, the changes in Klotho gene expression were accompanied by aggravation of renal lesions in ApoE KO mice, suggesting that Klotho could be involved in protection from hyperlipidemic renal damage. In line with this, renal Klotho expression was found to be downregulated in experimental models of CKD and AKI [18]–[20], [22] and in patients with CKD [21]. By contrast, *in vivo* Klotho gene delivery ameliorates renal damage and improves renal function in experimental models of kidney injury [20], [46].

Studies in hypercholesterolemic animal models showed that renal injury was accompanied by increased oxidative stress and inflammation [7], [8], changes that may decrease Klotho expression, as previously reported [22], [25], [26]. In our study ApoE KO mice fed HC displayed an inflammatory state characterized by enhanced macrophage infiltration, inflammatory chemokine expression (MCP-1 and RANTES) and oxidative stress in renal lesions. Increased kidney inflammation and oxidation, per se, could explain, at least in part, the lower Klotho expression observed in these hyperlipidemic animals. However the increased renal cholesterol content in mice fed HC diet and the general acceptance of the pathogenic role of LDL, particularly in its oxidized form, in renal injury [5]–[9], point to ox-LDL as a potential factor that may regulate Klotho expression in the hyperlipidemic state and may be situated upstream of inflammation.

Oxidized-LDL may affect the behavior of several renal cells types, including tubular epithelial cells [5], [6]. In pathological conditions, including CKD, renal tubular epithelial cells may be exposed to ox-LDL [5]. Oxidized modified lipoproteins have been identified in human kidney tissues [47] and are more abundant in plasma and kidneys from hyperlipidemic animals [48], [49]. Injurious actions of ox-LDL include induction of inflammation, oxidation and apoptosis, all of them processes associated with progression of renal disease [5], [50], [51]. Our *in vitro* study demonstrates that ox-LDL increased IL-6, RANTES, MCP-1 and TNF-α expression and secretion, and induced oxidative stress by increasing NADPH-oxidase mediated ROS production and intracellular superoxide anion levels. Interestingly, our data show for the first time that ox-LDL decreased both Klotho mRNA and protein expression in a time- and dose-dependent manner in cultured tubular epithelial cells. In agreement with our observations, previous studies reported that statin treatment, by decreasing cholesterol levels, significantly improved atherosclerotic lesions and vascular damage in rats via enhancing Klotho expression [24], [52].

The mechanism responsible for ox-LDL-induced Klotho down-regulation was also explored in the present study. It is known that ox-LDL mediate harmful effects through the activation of a variety of signaling pathways, including MAPK [35], ERK [36], protein kinase C [36], PI3K/Akt [36], and NF-κB [39]. However, the intracellular pathways regulating renal Klotho expression are less known. In a recent study, we demonstrated that the inflammatory cytokines TWEAK and TNF-α downregulate Klotho in renal tubular cells through an NF-κB-dependent mechanism [19]. Moreover, the RAS/MEK/ERK signaling cascade is involved in endothelial growth factor-induced activation of the Klotho promoter [53]. Here, we observed that ox-LDL promotes a rapid and transient activation of both ERK and NFκB signaling pathways in murine tubular cells. Moreover, blockade of the MEK-dependent pathway of ERK activation with PD98059 or blockade of NF-κB activation with parthenolide significantly reduced the biological effects of ox-LDL on Klotho expression, suggesting the involvement of both pathways in ox-LDL-induced Klotho decrease in tubular epithelium.

In conclusion, the present study reveals that hyperlipidemia induces inflammation, oxidative stress and accelerates renal damage in ApoE KO mice; and this is accompanied by down-regulation of Klotho expression. In addition, our data show that ox-LDL decrease Klotho expression in tubular cells through activation of ERK and NF-κB. These results suggest that Klotho may be a key element explaining the relationship between hyperlipidemia, aging and renal disease.

## Supporting Information

Figure S1 **Representative Oil-Red-O staining in C57BL/6 (WT) and ApoE knockout (ApoE) mice fed normal or hyperlipidemic diets (HC) for 10 weeks.** Note the glomerular lipid accumulation in ApoE KO mice, especially in those fed HC (arrows).
(TIF)

Figure S2 **Cell viability studies.** Proximal (MCT, **A**) and distal (NP-1, **B**) tubular epithelial cells were cultured for 24 hours in the presence of ox-LDL (0–25 µg/mL). Flow cytometry diagrams of permeabilized, propidium iodide–stained cells, showed no significant increase in apoptosis. Inset: Examples of nuclear morphology. Propidium iodide staining of permeabilized cells (original magnification ×800).
(TIF)

Figure S3 **Oxidized LDL decrease Klotho expression in cultured tubular distal cells.** Ox-LDL decreases Klotho mRNA expression, as determined by quantitative RT-PCR, in a time (**A**) and dose-dependent manner (**B**) in distal tubular epithelial cells (NP-1). Mean±SD of three independent experiments. *p<0.05 vs control. Klotho protein expression, as determined by Western blot (**C**) and confocal microscopy (**D**), in NP-1 treated with ox-LDL (25 µg/mL) for 24 h. Indirect immunofluorescence using anti-Klotho with secondary Alexa Fluor 488–conjugated antibody (green). Nuclei were stained with propidium iodide (PI, red). Images are representative of three independent experiments.
(TIF)
