# Supplementary figures and images for: Hyperlipidemia-Associated Renal Damage Decreases Klotho Expression in Kidneys from ApoE Knockout Mice
Source: PLoS One. 2013 Dec 30;8(12):e83713. doi: 10.1371/journal.pone.0083713 (PMC3875485; doi:10.1371/journal.pone.0083713)

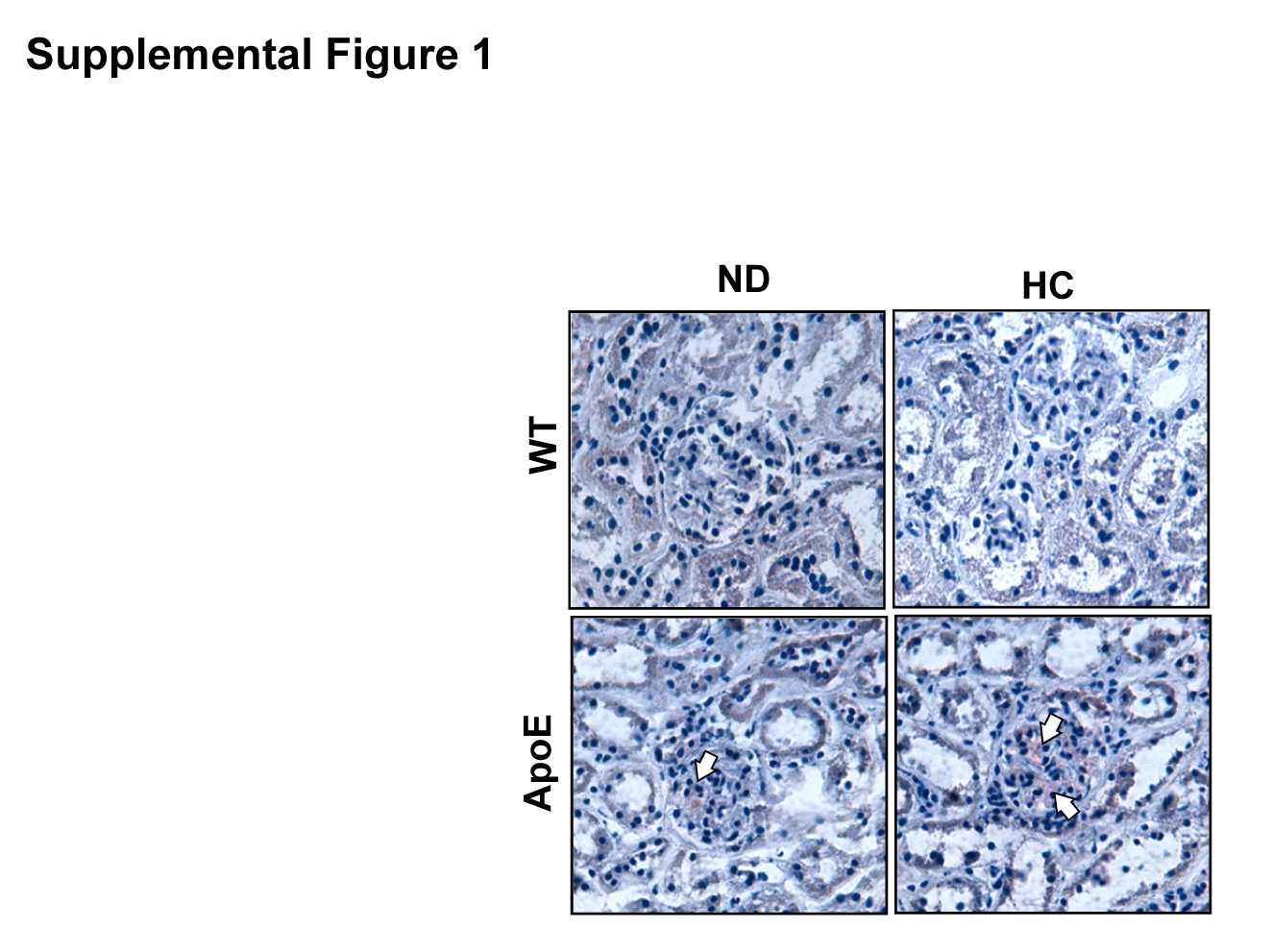

Supplement: Figure S1 — Representative Oil-Red-O staining in C57BL/6 (WT) and ApoE knockout (ApoE) mice fed normal or hyperlipidemic diets (HC) for 10 weeks. Note the glomerular lipid accumulation in ApoE KO mice, especially in those fed HC (arrows). (TIF) [file pone.0083713.s001.tif]

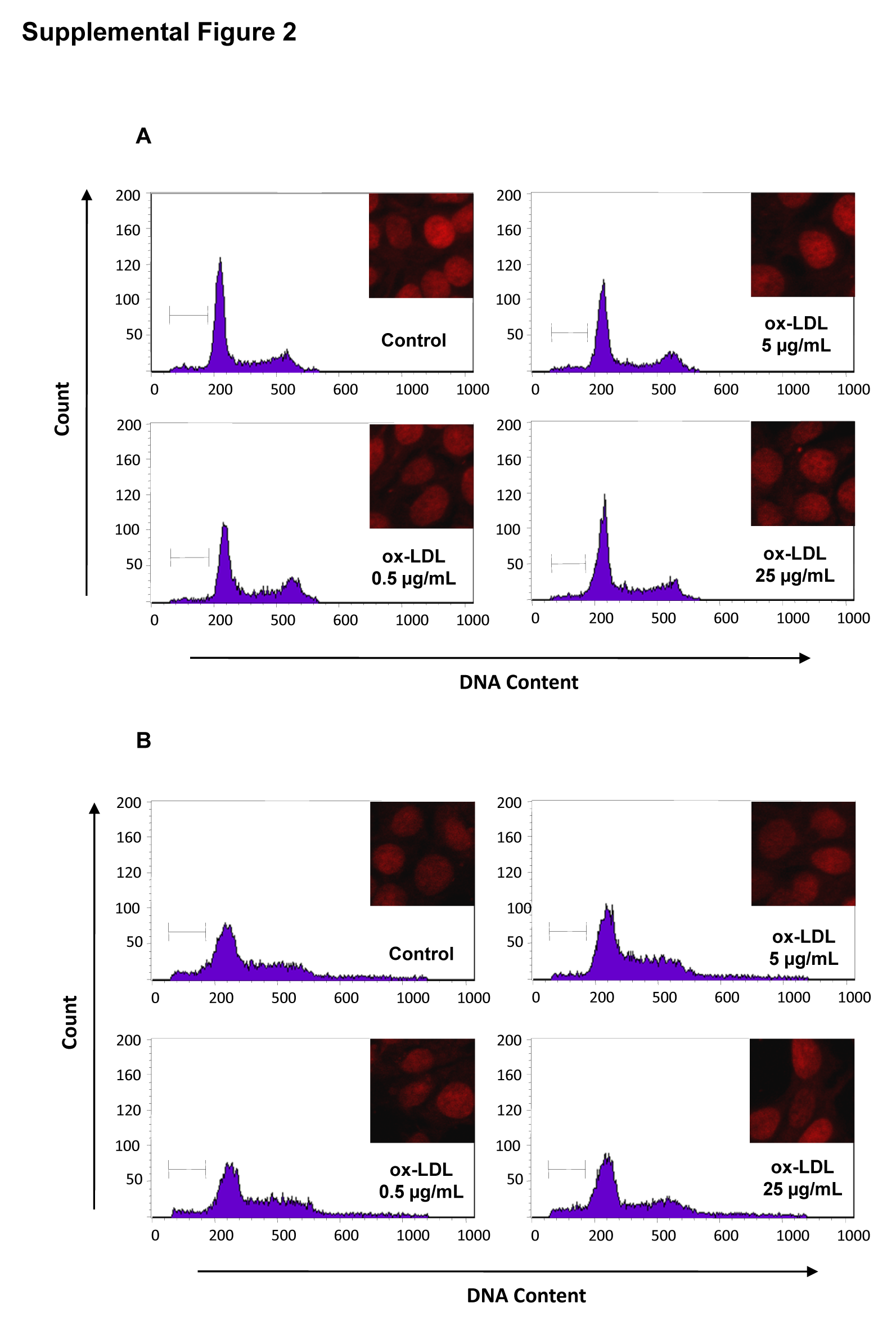

Supplement: Figure S2 — Cell viability studies. Proximal (MCT, A) and distal (NP-1, B) tubular epithelial cells were cultured for 24 hours in the presence of ox-LDL (0–25 µg/mL). Flow cytometry diagrams of permeabilized, propidium iodide–stained cells, showed no significant increase in apoptosis. Inset: Examples of nuclear morphology. Propidium iodide staining of permeabilized cells (original magnification ×800). (TIF) [file pone.0083713.s002.tif]

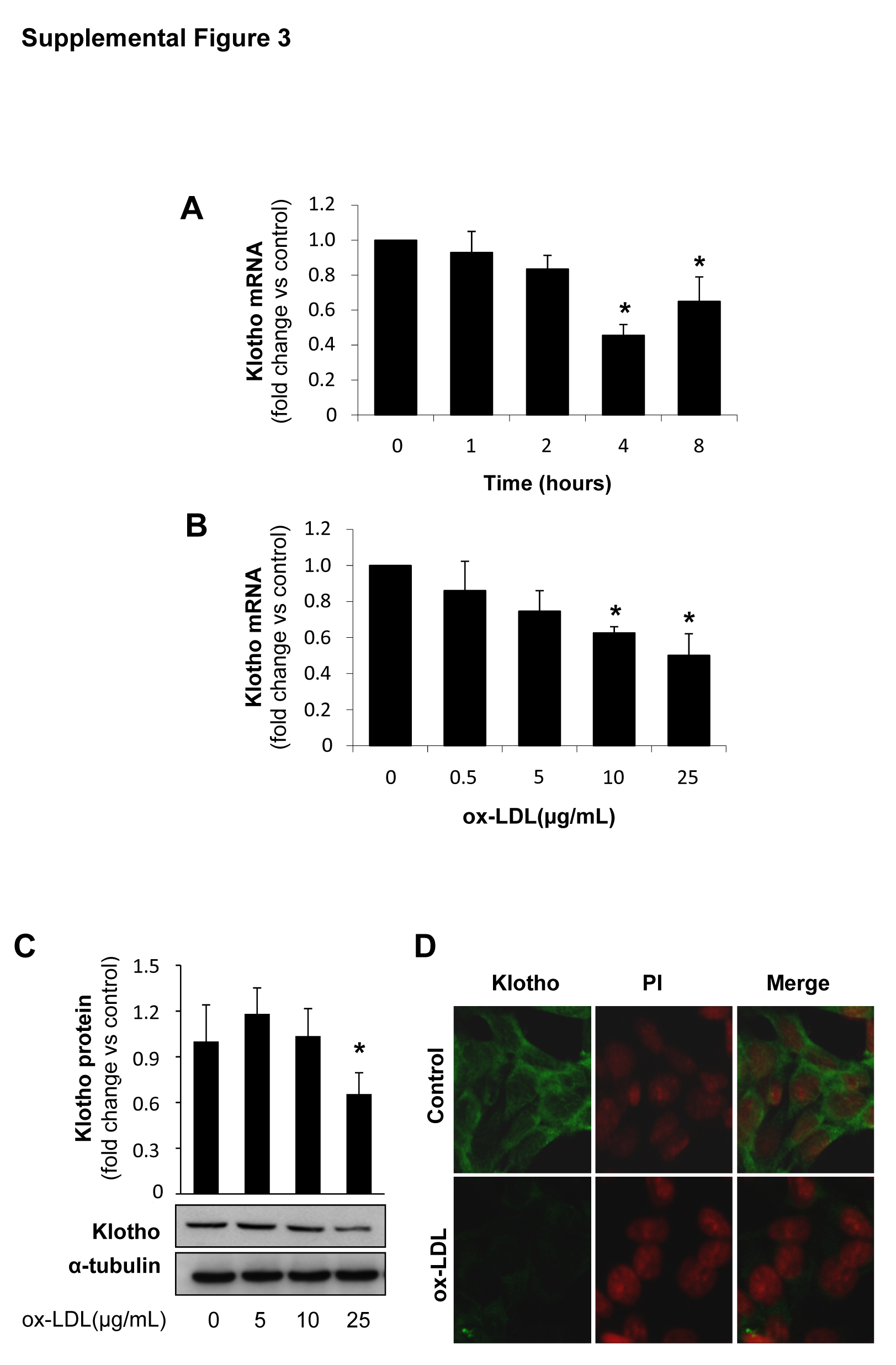

Supplement: Figure S3 — Oxidized LDL decrease Klotho expression in cultured tubular distal cells. Ox-LDL decreases Klotho mRNA expression, as determined by quantitative RT-PCR, in a time (A) and dose-dependent manner (B) in distal tubular epithelial cells (NP-1). Mean±SD of three independent experiments. *p<0.05 vs control. Klotho protein expression, as determined by Western blot (C) and confocal microscopy (D), in NP-1 treated with ox-LDL (25 µg/mL) for 24 h. Indirect immunofluorescence using anti-Klotho with secondary Alexa Fluor 488–conjugated antibody (green). Nuclei were stained with propidium iodide (PI, red). Images are representative of three independent experiments. (TIF) [file pone.0083713.s003.tif]
